# High-Intensity Aerobic Exercise Improves Both Hepatic Fat Content and Stiffness in Sedentary Obese Men with Nonalcoholic Fatty Liver Disease

**DOI:** 10.1038/srep43029

**Published:** 2017-02-22

**Authors:** Sechang Oh, Rina So, Takashi Shida, Tomoaki Matsuo, Bokun Kim, Kentaro Akiyama, Tomonori Isobe, Yoshikazu Okamoto, Kiyoji Tanaka, Junichi Shoda

**Affiliations:** 1 The Center of Sports Medicine and Health Sciences, Tsukuba University Hospital, Ibaraki, 305-8576, Japan; 2 Faculty of Medicine, University of Tsukuba, Ibaraki, 305-8575, Japan; 3 Japan Society for the Promotion of Science, Tokyo, 102-0083, Japan; 4 National Institute of Occupational Safety and Health, Kanagawa, 214-8585, Japan; 5 Graduate School of Comprehensive Human Sciences, University of Tsukuba, Ibaraki, 305-8575, Japan; 6 Faculty of Sports Health Care, Inje University, Gyeongsangnamdo, 50834, Republic of Korea; 7 Faculty of Health and Sport Sciences, University of Tsukuba, Ibaraki, 305-8575, Japan

## Abstract

We compared the effects of 12-week programs of resistance training (RT), high-intensity interval aerobic training (HIAT), and moderate-intensity continuous aerobic training (MICT). The primary goal was to evaluate the therapeutic effects of the exercise modalities for the management of nonalcoholic fatty liver disease (NAFLD). A total of 61 sedentary obese men with NAFLD were randomized into one of the following exercise regimens (RT, HIAT, or MICT). Hepatic fat content was decreased to a similar extent in the RT, HIAT, and MICT groups (−14.3% vs. −13.7% vs. −14.3%) without significant changes in weight and visceral fat. The gene expression levels of fatty acid synthesis were significantly decreased in the subjects’ monocytes. Hepatic stiffness was decreased only in the HIAT group (−16.8%). The stiffness change was associated with restored Kupffer cell phagocytic function (+17.8%) and decreased levels of inflammation such as leptin (−13.2%) and ferritin (−14.1%). RT, HIAT, and MICT were equally effective in reducing hepatic fat content, but only HIAT was effective in improving hepatic stiffness and restoring Kupffer cell function. These benefits appeared to be independent of detectable weight and visceral fat reductions; the benefits were acquired through the modulation of *in vivo* fatty acid metabolism and obesity-related inflammatory conditions.

Due to westernization of dietary habit and chronically decreased physical activity, the number of obese subjects is currently growing steadily. Visceral fat accumulation accompanied with obesity is an important disease background of nonalcoholic fatty liver disease (NAFLD)^1^,^2^. Since increased risks of cardiovascular events^3^ and diabetes mellitus have been observed in patients with NAFLD^4^, NAFLD should be regarded not only as a hepatic disease but also as a systemic disease.

Only dietary and exercise therapies have demonstrated effectiveness for the prevention of the onset and progression of NAFLD. Exercise is beneficial for reducing visceral fat and is reportedly effective in improving pathological conditions of NAFLD including fat accumulation, inflammation, and fibrosis^5^,^6^. The improvement and inhibition of the progression of these hepatic conditions by exercise can have a significant impact in the management of NAFLD.

Cross-sectional studies have shown that the hepatic pathological conditions of NAFLD are inversely correlated with the levels of physical activity^7^ and fitness^8^,^9^, which strongly support the direct benefit of exercise to the liver. Since the disease conditions of obesity are closely related to NAFLD^10^, management of NAFLD by dietary and exercise therapies is drawing more interest. Long-term studies are being conducted at many medical institutions to investigate the effect of exercise on fat accumulation in the liver and hepatic dysfunction and to reveal the effect of exercise separately from that of dietary therapy^6^,^11^. However, the evidence regarding the benefits of exercise to NAFLD is still insufficient.

A systematic review and meta-analysis were recently conducted to evaluate the efficacy of exercise interventions for the improvement in hepatic fat accumulation in NAFLD subjects^12^. The results indicated the benefits of exercise in decreasing hepatic fat accumulation even in cases of little or no weight reduction. Notably, reduced fat accumulation in the liver was observed with a level of exercise lower than the level that is currently recommended for the management of obesity^13^.

We reported that exercise alone improved hepatic dysfunction, even though exercise resulted in smaller changes in weight and visceral fat reduction than the results achieved with dietary restriction therapy. In addition, we conducted a study to determine the best exercise intensity and duration to reduce hepatic fat accumulation. Our results demonstrated that 250 min or more of moderate to vigorous physical activity each week had the greatest effect on hepatic fat reduction and its underlying pathophysiology, independent of weight reduction^14^.

Recently, the ameliorating effect of resistance exercise on hepatic fat accumulation in NAFLD has been observed^15^,^16^. Resistance exercise has only a small effect on weight reduction and body composition. However, resistance exercise has been demonstrated to increase basal energy expenditure by increasing muscle volume^17^, and improve insulin sensitivity^18^,^19^, a concurrent disease condition of obesity.

In addition to conventional exercise modalities, high-intensity interval aerobic training (HIAT) has recently been introduced as a new exercise therapy alternative^20^. In HIAT, short duration, high-intensity aerobic exercise and recovery time with low-load aerobic exercise are alternately repeated^20^. HIAT can be completed in 13 min. HIAT is thought to be the most effective form of exercise for subjects who do not have much time. The benefits of HIAT on cardiovascular disease^21^ and metabolic syndrome^22^ have also been reported.

We sought to establish evidence for the most appropriate modality, intensity, time, and duration of exercise for the management of NAFLD. Therefore, in this study, we conducted a randomized controlled trial (RCT) to compare the effects of 12 weeks of resistance training (RT), HIAT, and moderate-intensity continuous aerobic training (MICT) in obese men with NAFLD. By analyzing the participants’ baseline and follow-up clinical measurements, we comparatively investigated the effects of the different exercise modalities on NAFLD.

## Methods

### Ethical approval

These clinical trials were approved by the Institutional Review Board of University of Tsukuba Hospital (ID: H25–156) and retrospectively registered with the University Hospital Medical Information Network Clinical Trials Registry (UMIN-CTR ID: UMIN000022901). All the procedures were carried out in accordance with the principles of the Declaration of Helsinki. We fully explained the purpose and design of the study to all the participants, and each participant signed an informed consent document.

### Study Design

**Figure 1:**
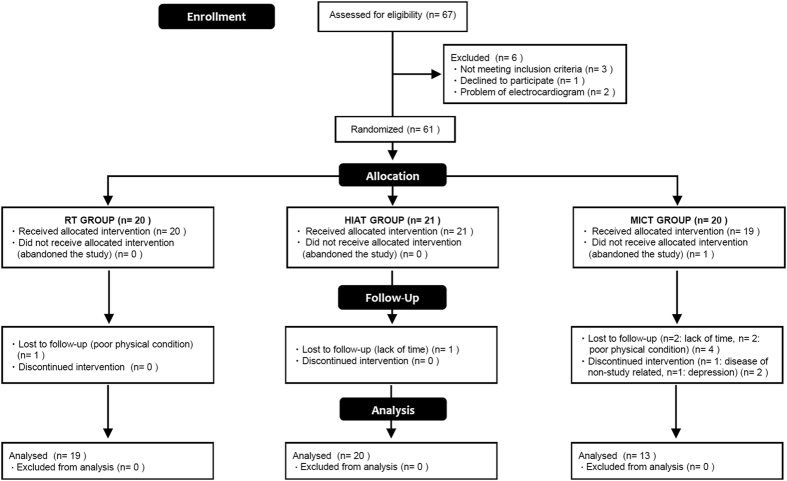
Flowchart showing the study process. RT, resistance training; HIAT, high intensity aerobic training; MICT, moderate intensity continuous training.

The workflow of enrollment to the program and exclusion criteria is provided in Fig. 1.

The participant recruitment, 12-week study intervention, and clinical tests were all performed at University of Tsukuba Hospital (Ibaraki, Japan) in 2013. Out of the initial 67 male applicants, 61 obese^23^ sedentary adult men with NAFLD were enrolled according to the study criteria. Obese adult men with no exercise habits (≤1 session per week and ≤30 min per session) over the past year were included. Also, subjects with adverse medical problems (all applicants undertook a medical interview and resting electrocardiogram test by a medical doctor) and who declined to participate in the current protocol were excluded. The diagnostic criteria of NAFLD were established by the diagnostic guidelines for NAFLD in the Asia-Pacific region^24^.

We conducted a prospective, single blind RCT for this study. After obtaining the baseline measurements, the 61 eligible subjects were assigned in a 1:1:1 ratio to one of the three intervention groups by a computerized method (EXCEL 2010; Microsoft Corp, Redmond, USA) using block randomization with stratification on age and VO_2Max_. A research assistant who had no interaction with the subjects generated the random allocation sequence and enrolled the subjects. Out of the 61 subjects who were assigned to the exercise training programs consisting of 12 weeks, 3 times/week from August to December 2013, a total of 52 subjects (RT [n = 19] vs. HIAT [n = 20] vs. MICT [n = 13]) completed the study.

### One Repetition Maximum (1-RM) Strength and Cardiorespiratory Capacity Test

The subjects in the RT group were given a 1-RM strength test. After a learning phase, the subjects performed 3 series of 12 repetitions at a relatively light load as a warm-up. The warm-up was followed by a gradually increasing load until they achieved the 1-RM strength within 5 attempts with 1 min of recovery between series. We conducted the tests taking into consideration assisting the recovery and reducing the influence of fatigue. The tests were alternated between the upper and lower muscles.

The VO_2Max_ test in the aerobics groups (HIAT and MICT) was performed on a graded direct cycling ergometer [75XL, Konami, Tokyo, Japan] at weeks 0, 4, 8, and 12. Following a 2-minute warm-up at 30 watts, the workload increased every minute by 15 watts until volitional exhaustion. During the test, ventilation and gas exchanges were measured using an open-circuit computerized indirect calorimeter [AE-310S, Minato Medical Science, Osaka, Japan]. Heart rate at rest and during the test was supervised using an electrocardiogram monitor [DynaScope, Fukuda Denshi, Tokyo, Japan].

### Training Programs

All subjects performed their assigned training program three times per week on nonconsecutive days for 12 weeks. During the study period, all of the subjects were asked not to perform the trial-specific exercise activities outside of the training program. Also, we asked each subject to not change anything about their lifestyle and diet for 12 weeks.

The RT program referred to the ACSM 2009 position paper on “Progression Models in Resistance Training for Healthy Adults”^25^. The program consisted of 1) sit-ups, 2) leg presses, 3) leg extensions, 4) leg curls, 5) chest presses, 6) seated rows, and 7) pull-downs (Selection MED, Technogym, Cesena, Italy). The amount of load lifted was updated according to the results of the monthly direct 1-RM strength test. The total energy expenditure for the RT program was estimated to be about 180 kcal in our preliminary experiment. These values are similar with HIAT.

The detailed descriptions of the aerobics training (HIAT and MICT) regimens have been published elsewhere^20^,^26^. Briefly, the HIAT consisted of three sets of 3-min cycling sessions at 80~85% VO_2Max_ with a 2-min active rest at 50% VO_2Max_ between sets (13 min, 180 kcal), and the MICT consisted of 40 min. of cycling at 60~65% VO_2Max_ (40 min, 360 kcal). The exercise intensity was recalculated and updated following the monthly VO_2Max_ measurements recording.

### Daily Energy Intake

At baseline and at week 12, daily energy intake was estimated using both three-day and weight dietary records. The study subjects photographed and recorded the name and amount of every food item they ate. A dietician analyzed the dietary data using commercially available computer software [Eiyoukun version 6.0, Kenpakusya, Tokyo, Japan].

### Anthropometry and Body Adiposity

Body weight was measured to the nearest 0.05 kg using a digital scale [WB-150, TANITA, Tokyo, Japan], and height was measured once to the nearest 0.1 cm using a wall-mounted stadiometer [YG-200, Yagami, Nagoya, Japan]. BMI was calculated as the weight divided by height squared (kg/m^2^). The study participants’ body composition was evaluated by dual-energy x-ray absorptiometry using a total body scanner [QDR 4500, Hologic Inc, Bedford, USA]. Their abdominal distribution was determined using magnetic resonance imaging [Achieva, Philips Electronics Japan Ltd, Tokyo, Japan], according to a previously described protocol^27^. Individual’s adipose tissue volume was calculated by multiplying the subcutaneous adipose tissue (SAT) and visceral adipose tissue (VAT) areas at the umbilicus level.

### Hepatic Stiffness and Steatosis

Hepatic stiffness was assessed using transient elastography [FibroScan502^®^, Echosens, Paris, France] with the 3.5-MH_z_ standard probe by a clinical gastroenterologist. The principles and examination procedures for such an assessment have been previously published^28^.

Hepatic fat content was determined using a controlled attenuation parameter (CAP) designed to measure the liver ultrasonic attenuation at 3.5 MH_z_ using signals acquired with FibroScan502^®^. More detailed descriptions of the CAP have also been previously published^29^. Moreover, intrahepatic fat accumulation was determined by proton magnetic resonance spectroscopy [Achieva, Philips Electronics Japan Ltd, Tokyo, Japan] using a previously described protocol^30^.

### Contrast Enhanced Ultrasonography (CEUS) of Liver

The CEUS liver parenchymal phase was used to determine the phagocytic capacity of Kupffer cells^31^. The detailed protocol for imaging the liver through contrast ultrasonography has been published elsewhere^32^. This time, we applied this protocol with some modification for the current CEUS measurements. Briefly, sonazoid used as a contrast agent was diluted to 0.1 mL/1200 g/body weight and injected into the vein of the subjects. A clinical gastroenterologist scanned the liver of the subjects using 1-second intermittent transmission scans at 40 min using ultrasonography [Aplio 400, Toshiba medical, Tokyo, Japan]. The fluorescent ROI [intensity in the region of interest] was calculated using the equipment software Advanced Dynamic Flow [Toshiba medical, Tokyo, Japan].

### Blood Analysis

The level of fasting plasma glucose (FPG) was determined by the hexokinase-G-6-PDH method; fasting plasma insulin (FPI) by the chemiluminescent immunoassay method; aspartate aminotransferase (AST), alanine aminotransferase (ALT), and gamma glutamyl transpeptidase (γGT) by the Japan Society of Clinical Chemistry transferable method; triglyceride; and free fatty acids (FFAs) by the enzymatic method. We calculated surrogate markers for insulin resistance (HOMA-IR)^33^ and for the NAFLD fibrosis score (NAFLD-FS)^34^. Commercial ELISA and ECL assay kits were used to determine serum levels of thiobarbituric acid reactive substances (TBARS) [Cayman Chemical, Ann Arbor, USA], tumor necrosis factor alpha (TNF-α), interleukin-6 (IL-6), fibroblast growth factor-21 and leptin [R&D systems, Minneapolis, USA], total adiponectin [Sekisui Medical, Tokyo, Japan], M30 apoptosense [Pavia, Bromma, Sweden], Selenoprotein P (SEPP1) and Myostatin (MSTN) [Cusabio biotech, Wuhan, China], Fetuin A [BioVendor Laboratory Medicine, Modreci, Czech Republic], WFA^+^ -Mac-2 binding protein (WFA^+^ -Mac M2BP) [Immuno-Biological Lab, Kunma, Japan]. Serum lipopolysaccharide (LPS) concentration was determined using the Limulus amoebocyte lysate assay kit [Associates of Cape Cod, East Falmouth, USA].

### Quantitative Real-Time PCR analysis

Gene levels in peripheral blood mononuclear cells (PBMCs) are known as a good model to reflect physiological changes in the liver^35^, since liver and PBMCs originate evolutionarily from the same body compartment^36^ PBMCs were isolated from whole blood using LSM density gradients [MP Biomedical, Santa Ana, USA]. The mRNA levels in the PBMCs were analyzed by real-time quantitative PCR using recently detailed methodology^14^. The primers (Fasmac, Tokyo, Japan) used in this study are shown in Table 1.

### Statistical Analysis

Descriptive values were expressed as the means ± standard error (SE) and as percentages. For the analysis of categorical parameters, the chi-squared test or Fisher exact test was performed. To examine the differences among the groups at the baseline we used the one-way analysis of variance (ANOVA) test. Paired *t* tests were performed to test the significance of changes in clinical parameters within the groups. We also compared parameters among the groups that changed from the baseline to the 12^th^ week using either the one-way ANOVA test or the analysis of covariance (ANCOVA) test with adjustments for the respective baseline values. The data were analyzed using SPSS version 23.0 for Windows package [IBM, Chicago, USA]. The level of statistical significance was set at *P* < 0.05.

## Results

### Baseline Characteristic

**Figure 2:**
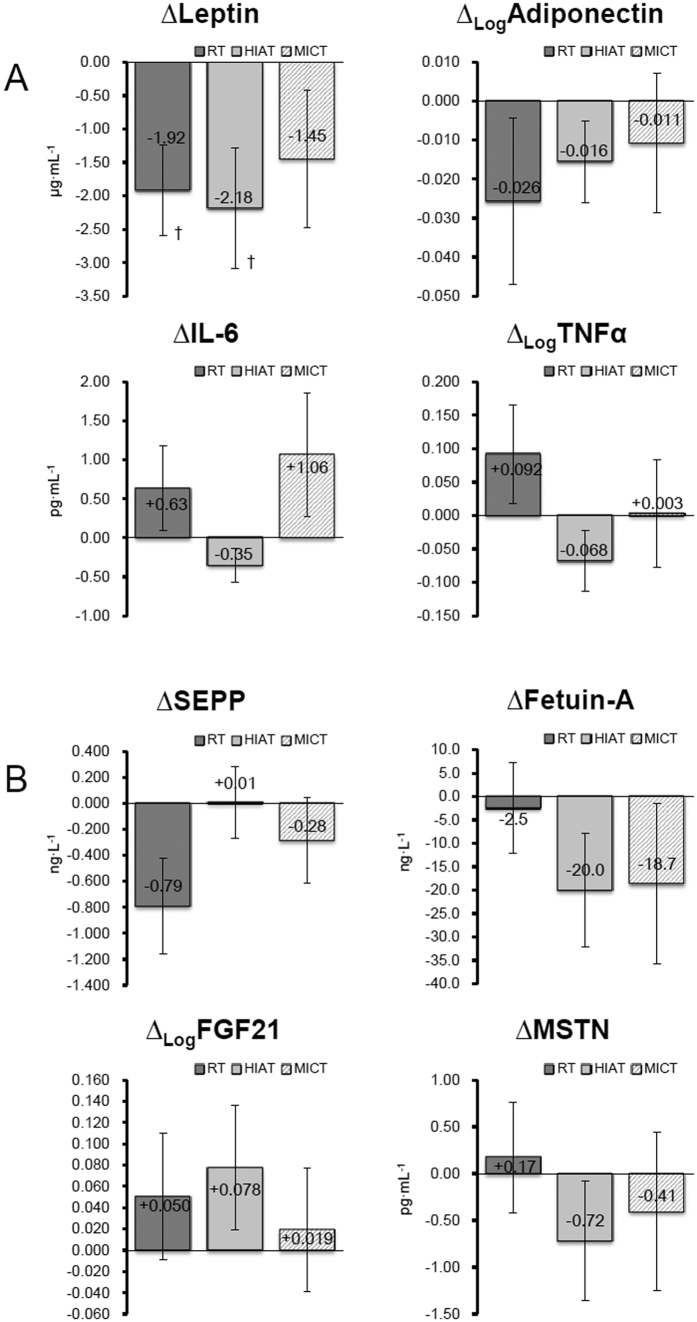
Changes in the levels of adipokine (**A**), hepatokine and myokine (**B**) from the baseline to the end point of 12 weeks in a total of 52 subjects (RT = 19, HIAT = 20 and MICT = 13) with NAFLD who were randomly allocated to a 12-week training program. Analysis of SEPP used ANCOVA adjusted for baseline values to compare changed values between groups. ^†^*P* < 0.05, significant difference between the baseline and the 12^th^ week. IL-6, interleukin 6; TNF-α, tumor necrosis factor alpha; SEPP1, selenoprotein P; FGF-21, fibroblast growth factor 21; MSTN, myostatin.

There were no statistically significant differences in the subjects’ characteristics and use of medications among the 3 groups (Table 2). The mean energy intake, anthropometric values (Table 3), and the hepatic steatosis and liver fibrosis marker levels (Fig. 2) were not significantly different among the groups. However, there were statistical differences in the 6 baseline serum values of _Log_AST (*P* < 0.05), ALT (*P* < 0.05), _Log_FPG (*P* < 0.01), HOMA-IR (*P* < 0.05), _Log_M30 (*P* < 0.01) and SEPP (*P* < 0.05) among the groups (Table 4). The gene expression levels (Table 5) were not significantly different among the 3 groups.

### Intervention Adherence

Table 3 shows the results of energy intake for each group during the study period. According to the 3-d food-intake records, the 3 groups showed no significant changes in their energy intake values at week 12. The attendance rates were 89.5% in the RT group, 92.1% in the HIAT group, and 92.1% in the MICT group. The differences in attendance were not statistically significant (data not shown).

### Body Weight and Cardiovascular Capacity

There were no significant changes in weight at week 12 in any of the groups, and the magnitude of the changes in weight were not significantly different among the groups (Table 3). The subjects’ VO_2_max was increased in all 3 groups (RT + 8.6%, HIAT + 21.6%, MICT + 16.1%) when the baseline results were compared to the week 12 results. A comparison among the 3 groups revealed that the changes were not statistically significant (data not shown).

### Body Adiposity

The subjects’ fat mass was reduced in the RT and MICT groups, and lean mass was increased in the RT and HIAT groups when the baseline results were compared to the week 12 results (Table 3). However, the 2 measured values for abdominal distribution were not significantly changed at week 12. When an intergroup comparison was made, the magnitude of the change in these values was not significantly different.

### Blood Test

**Figure 3:**
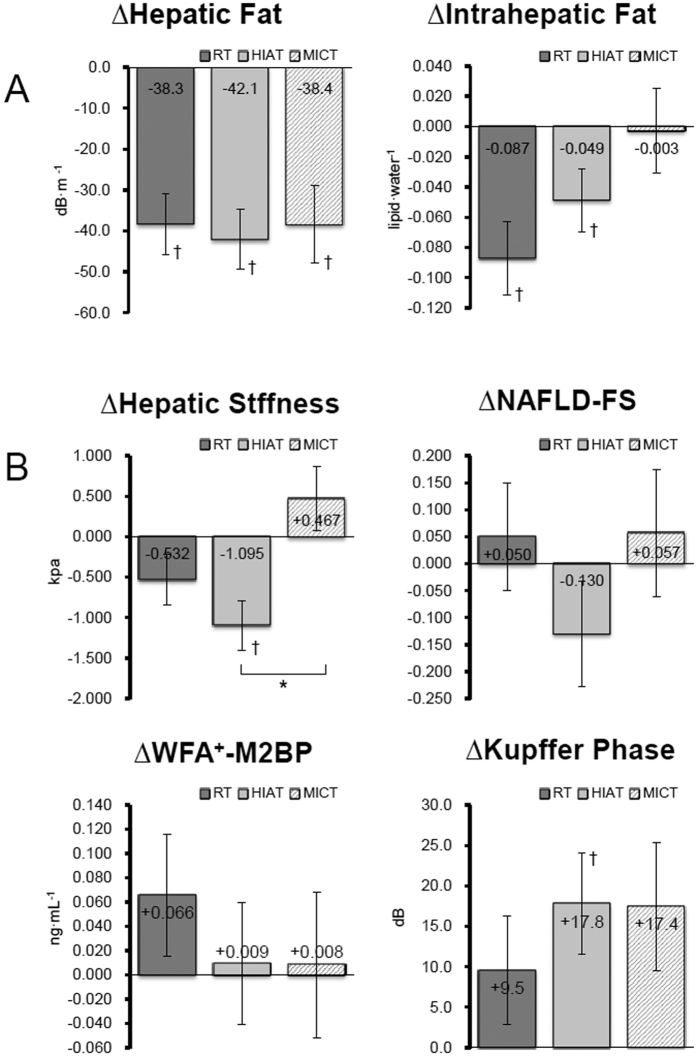
Changes in the levels of hepatic steatosis (**A**) and markers associated with liver fibrosis (**B**) from the baseline to the end point of 12 weeks in a total of 52 subjects (RT = 19, HIAT = 20 and MICT = 13) with NAFLD who were randomly allocated to a 12-week training program. ^†^*P* < 0.05, significant difference between baseline and 12^th^ week; brackets *P < 0.05, significant difference among the three program groups. NAFLD-FS, non-alcoholic fatty liver disease fibrosis score; WFA^+^ -M2BP, wisteria floribunda agglutinin-positive human Mac-2-binding protein.

Of the 11 values in biochemical values that were analyzed in this study (Table 4), three values (_Log_γ-GTP, FFAs and LPS) in the RT group (*P* < 0.05), two values (_Log_γ-GTP and Ferritin) in the HIAT group (*P* < 0.05), and four values (ALT, _Log_γ-GTP, FFAs and LPS) in the MICT group (*P* < 0.05) were significantly changed at week 12. The comparison among the groups revealed that the magnitude of the changes in all the values were not statistically significant. With regard to the markers associated with liver fibrosis, WFA^+^ -M2BP (Fig. 3B), there was no significant change in these values in any of the training groups at 12 weeks. Also, when an intergroup comparison was made, the magnitude of the changes in the WFA^+^ -M2BP values among the groups was not significantly different. For the adipokine, myokine and hepatokine analysis (Fig. 2), the RT group (−14.3%) and the HIAT group (−13.2%) showed significantly decreased levels of leptin at week 12 (*P* < 0.05).

### Hepatic Steatosis

The subjects’ hepatic fat content, as assessed by CAP, was reduced in all 3 groups (RT −14.3%, HIAT −13.7%, MICT −14.3%), when the baseline results were compared to the week 12 results (*P* < 0.05). However, a reduction in the intrahepatic fat content by MRS was only detected in the RT group (−47.2%) and the HIAT group (−16.6%) at week 12 (*P* < 0.05); the MICT group did not demonstrate any significant change in that value. A comparison among the 3 groups revealed that the changes were not statistically significant (Fig. 3A).

### Liver Stiffness and Associated Markers

Figure 3(B) shows the results of changes in the hepatic stiffness assessed using transient elastography, NAFLD-FS as a surrogate marker, WFA^+^ -M2BP as a blood marker for hepatic stiffness, and the Kupffer phase assessed by contrast ultrasonography, for each group during the study period. In the HIAT group, significant improvements were observed in hepatic stiffness by −16.8% and in the Kupffer phase by +17.8% (*P* < 0.05). However, the other groups did not demonstrate significant changes. When an intergroup comparison was made, the magnitude of change in hepatic stiffness was greater in the HIAT group than in the MICT group (*P* < 0.01).

### Expression Levels in the PBMCs

Table 5 shows the changes in the expression levels in the PBMC of the five fat metabolism-related genes (fatty acid synthesis: _Log_SREBP1c [sterol regulatory element-binding protein 1c], _Log_ACC [acetyl-CoA carboxylase]; fatty acid degradation: _Log_CPT-1 [carnitine palmitoyltransferase-1], _Log_ACO [acyl-CoA oxidase], five macrophage specific genes (_Log_CD11b [cluster of differentiation 11b], _Log_CD14 [cluster of differentiation 14], _Log_CD68 [cluster of differentiation 68], _Log_TLR4 [toll-like receptor 4], _Log_TLR5 [toll-like receptor 5], two nuclear factor E2–related factor 2 (Nrf2) target genes (_Log_HO1 [heme oxygenase], _Log_NQO1 [NADH quinone oxidoreductase]) in each group after 12 weeks. Among the eleven genes, eight (except _Log_CPT-1, _Log_HO-1 and _Log_NQO1) in the RT group (*P* < 0.05), nine (except _Log_CPT-1 and _Log_NQO1) in the HIAT group (*P* < 0.05), and nine (except _Log_HO-1 and _Log_NQO1) in the MICT group revealed significant changes (*P* < 0.05). However, a comparison among the groups revealed that the magnitude of the changes in all these measurements was not significantly different.

## Discussion

A need of alternative strategies with increased exercise or physical activity is strongly emphasized in the management of NAFLD. We conducted a prospective randomized controlled study and comparatively investigated the effects of the differences in modality and intensity of exercise to make an appropriate proposal in the management of NAFLD.

The important findings of this study are as follows:

1. All three modalities of exercise, RT, HIAT, and MICT, resulted in a similar degree of improvement in the hepatic fat content. The modality and intensity of the exercises did not appear to be important factors.
2. Improvements in hepatic stiffness were only observed in the HIAT group. In the HIAT group, improvements were also detected in the phagocytic function of Kupffer cells for foreign bodies. The intensity of exercise did appear to be an important factor in the improvements in hepatic fibrosis and inflammation.
3. The improvements in hepatic fat content and stiffness were not related to the reductions in weight or visceral fat. Exercise may directly benefit the pathophysiological conditions of NAFLD.

In this study, increased VO_2Max_, a cardiopulmonary capacity, was observed in all three groups with RT, HIAT, and MICT, showing the effect of 12-week training. Despite differences in the training approach, the degree of improvement in hepatic fat content by training of RT, HIAT, and MICT was equivalent without any significant difference among the three groups. The measurement in hepatic fat content was performed with the CAP on Transient Elastography. It has been demonstrated that the measured values of CAP are highly correlated with the levels of fat deposition in tissue specimens of the liver^37^. CAP has a high sensitivity even in patients with mild hepatic steatosis and is a useful diagnostic tool for NAFLD^29^. Based on the above, it is presumed that the modality and intensity of exercise do not play an important role in the improvement in hepatic fat content through exercise.

In obese subjects with NAFLD, FFAs derived from visceral adipose tissues are the major cause of hepatic fat synthesis^38^. Accordingly, it can be assumed that reducing the inflow of FFAs derived from adipose tissues into the liver will reduce hepatic fat accumulation in patients with NAFLD.

A decrease in the serum levels of FFAs was observed in all 3 groups, which suggests that there was a reduction in the inflow of FFAs into the liver. This observation may be attributed to the exercise-induced recovery of insulin sensitivity in the adipose tissues, which would in turn inhibit lipolysis in the tissues, leading to the reduction in the levels of FFAs^38^. These functional changes in the adipose tissues, rather than a reduction in the adipose tissue volume, may have been important since the measured changes in body weight and composition were very small (Table 3).

Moreover, in our analysis of metabolic dynamics using PBMCs, the reduced expression levels of SREBC-1c and ACC, a transcription factor that regulates fatty acid synthesis, was observed in all 3 groups; therefore, we presumed that *de novo* fatty acid synthesis was reduced in the liver with exercise. In contrast, no increase was observed in the expression levels of CPT-1 and ACO, which are involved in beta-oxidization in fatty acid degradation. This finding suggests that the modality and intensity of the training regimens reduced hepatic fat accumulation through modulation of fatty acid synthesis in the liver, regardless of the exercise type (Table 5).

In this study, HIAT, which is a high-intensity, low-volume, short-time exercise with lower energy consumption, showed a significantly better effect on hepatic stiffness and the phagocytic function of Kupffer cells. This finding is notable for the management of NAFLD (Fig. 3).

Tissue diagnosis by liver biopsy is required for a definite diagnosis of NASH. However, non-invasive diagnostic approaches were recently developed and the benefits on diagnoses of hepatic steatosis and fibrosis on contrast-enhanced ultrasonography or elastography have been reported^32^,^39^. The measurement of hepatic stiffness by transient elastography is considered to precisely reflect fibrosis in the liver tissue samples^40^,^41^. In addition to fibrosis, other factors that affect hepatic stiffness include inflammation, cholestasis, and venous pressure^42^. An important consideration in the management of NAFLD is that HIAT ameliorates fibrotic and inflammatory conditions of the liver. Although improvements in hepatic fat accumulation due to exercise have been reported in many studies of NAFLD^5^,^6^, there have been few studies that examined improvements in hepatic fibrosis and inflammation^43^,^44^.

Research has determined that the phagocytic activity of Kupffer cells is reduced in the livers of nonalcoholic steatohepatitis (NASH) patients^45^,^46^. Rencently, it has been revealed that Sonazoid, an intravenous contrast-enhanced agent for ultrasonography, is phagocytized by Kupffer cells and that phagocytic activity of Kupffer cells is reduced in NASH^45^. Reduced uptake of contrast-enhanced agent into the liver is observed in the early stage of NASH^46^. In our study, the luminance level of the Kupffer cell-phase was remarkably reduced in moderately to severely obese subjects with suspicious liver fibrosis (Findings submitted elsewhere for publication). It is likely that HIAT restored the impaired Kupffer cells’ phagocytic function, which in turn improved the hepatic inflammation and fibrogenesis associated with NAFLD.

In the HIAT group, the levels of ferritin, an inflammatory marker (Table 4), and the levels of leptin, an adipokine that induces inflammatory reactions and hepatic fibrosis, were significantly reduced (Fig. 2) after the training. Gene expressions of inflammatory mediators TLR4, TLR5, CD11b, and CD14 were reduced in the PBMCs. These results were consistent with previous studies^47^,^48^ that have demonstrated that exercise exerts anti-inflammatory effects through the decreased expression of TLR4. Our results revealed a further increase in serum LPS concentrations induced by the exercise training. This suggests an increased flux of LPS from the intestines. It is likely that in the obese subjects in the HIAT group, the decreased TLR4/CD14 expression contributed to the attenuation of LPS-induced inflammatory reactions in the liver. In addition, the restored phagocytic function of Kupffer cells for foreign bodies likely contributed to the further attenuation.

Another possible explanation for the improvement in the inflammation was Nrf2 activation induced by the high-intensity exercise^49^. Recently, researchers have reported that Nrf2 restrains macrophage inflammatory response through opposing transcriptional upregulation of the proinflammatory cytokine gene^50^. HIAT considerably increased the expression level of HO-1, which is known to be a prototypical Nrf2 target gene (Table 5). Collectively, the changes in the expression levels of these molecules induced by HIAT would be expected to improve *in vivo* inflammatory conditions in obese men and lead to improvements in hepatic stiffness.

The findings of Kistler *et al*. support our observations. Kistler *et al*.^43^ conducted an investigation of the relationship between the intensity of exercise and the histological severity of NAFLD. Their results revealed that high-intensity physical activity was closely connected with reduced adjusted odds of morbidity due to NASH. They concluded that the intensity of exercise was an important factor in the improvements in hepatic inflammation and fibrosis.

This study has the strength of being a prospective randomized controlled study. Obese adult men with NAFLD were randomly allocated to one of three groups (RT, HIAT, and MICT group). The exercise training was conducted under the supervision of experts, and state-of-the-art techniques for the assessment of features were used as the analytical methods. On the other hand, this research is limited in that no sedentary control group was set. No liver biopsy was performed because of ethical issues, and no histological evaluation on steatosis and fibrosis was made. In particular, the correlation between exercise training and the pathological conditions of the liver tissues (inflammation and fibrosis) was not revealed. Finally, our study design did not consider sampling size calculation to estimate the effect of sample size. Therefore, the small sample size might have limited the statistical power of the study.

In summary, the results of a prospective randomized controlled study suggest that 12-week exercise regimens of RT, HIAT, and MICT all improved hepatic fat accumulation, independent of reduction of weight or visceral fat. HIAT also appeared to improve hepatic stiffness, and thus this modality could be recommended for NAFLD subjects with suspected fibrosis. It should be noted that exercise was beneficial for the management of NAFLD even when conducted without concomitant dietary therapy. In the future, it will be necessary to further clarify the role of exercise training in the management of NAFLD in a large-scale clinical study.

## Additional Information

**How to cite this article**: Oh, S. *et al*. High-Intensity Aerobic Exercise Improves Both Hepatic Fat Content and Stiffness in Sedentary Obese Men with Nonalcoholic Fatty Liver Disease. *Sci. Rep.* **7**, 43029; doi: 10.1038/srep43029 (2017).

**Publisher's note:** Springer Nature remains neutral with regard to jurisdictional claims in published maps and institutional affiliations.

## Figures and Tables

**Table 1:** Primers Used for Quantitative Real-Time PCR.

Gene name	Forward	Reverse
GAPDH	5′-AGGTGAAGGTCGGAGTCA-3′	5′-GGTCATTGATGGCAACAA-3′
SREBP1c	5′-ATACCACCAGCGTCTACC-3′	5′-CACCAACAGCCCATTGAG-3′
ACC	5′-ATGTCTGGCTTGCACCTAGTA-3′	5′-CCCCAAAGCGAGTAACAAATTCT-3′
CPT1	5′-CTGTGCGCCCCTTGTTGGATG-3′	5′-GGGCTGCCTGCACGTCTGTATT-3′
ACO	5′-AATCGGGACCCATAAGCCTTT-3′	5′-GGGAATACGATGGTTGTCCATTT-3′
CD11b	5′-GAGTCCAACGCTAATGTCAAGG-3′	5′-CCCGTAGAGAACAGCATCACAC-3′
CD14	5′-GAGTGTGCTTGGGCAATGCT-3′	5′-ATGCTGACACGGTCAAGGCT-3′
CD68	5′-ATGATGAGAGGCAGCAAGATGG-3′	5′-GCTACATGGCGGTGGAGTACAA-3′
TLR4	5′-CTAAACCAGCCAGACCTTG-3′	5′-ACCTGTCCCTGAACCCTAT-3′
HO1	5′- CCAGGCAGAGAATGCTGAGT-3′	5′-GTAGACAGGGGCGAAGACTG-3′
NQO1	5′-CTGATCGTACTGGCTCACTC-3′	5′-AACAGACTCGGCAGGATAC-3′

Abbreviations: GAPDH, glyceraldehyde 3-phosphate dehydrogenase; SREBP1c, sterol regulatory element-binding protein 1c; ACC, acetyl-CoA carboxylase; CPT1, carnitine palmitoyltransferase I; ACO, acyl CoA oxidase; CD, cluster of differentiation; TLR, toll-like receptor; HO1, heme oxygenase 1; NQO1, NADH quinone oxidoreductase.

**Table 2:** Baseline Characteristics of 52 Subjects with NAFLD Who were Randomly Allocated to One of Three 12-week Exercise Training Programs.

	RT	HIAT	MICT	*P*
	n = 19	n = 20	n = 13	
**Demographic characteristics**				
Age, years	51.2 ± 1.9	48.6 ± 1.8	48.2 ± 2.3	0.517
BMI, kg·m^−2^	27.2 ± 0.9	28.4 ± 0.9	28.8 ± 1.1	0.470
Body height, cm	172.6 ± 1.6	170.7 ± 1.0	171.2 ± 1.8	0.618
Waist circumference, cm	95.3 ± 1.2	97.1 ± 1.6	100.1 ± 4.0	0.336
**Medications**				
Hyperglycemic, %	5.3	0	0	0.404
Hypertensive, %	26.3	30.0	0	0.138
Hyperlipidemic, %	10.5	15.0	0	0.337
Smoking, %	36.8	30.0	38.5	0.878
**Cardiovascular Capacity**				
VO_2_max, ml/kg/min	29.1 ± 1.3	29.2 ± 1.2	29.1 ± 1.5	0.998

Values are presented as the group means ± SE and as percentages. Abbreviations: RT, the group that performed resistance training; HIAT, the group that performed high-intensity interval aerobic training; MICT, the group that performed moderate-intensity continuous training; BMI, body mass index; VO2max, maximal oxygen consumption.

**Table 3:** Energy Intake and Anthropometry Values in 52 Subjects with NAFLD Who were Randomly Allocated to One of Three 12-week Exercise Training Programs.

	RT			HIAT			MICT			*P*
	n = 19			n = 20			n = 13			
	Baseline	After	Change	Baseline	After	Change	Baseline	After	Change	
**Energy Intake**										
TEI, kg/d^−1^	1983 ± 85	1868 ± 86	−115	1929 ± 87	1905 ± 100	−24	2041 ± 211	2061 ± 89	+20	0.849
Carbo, g/d^−1^	252.1 ± 14.1	233.8 ± 12.1	−18.3	262.5 ± 14.3	271.9 ± 15.1	+9.4	274.9 ± 31.8	272.7 ± 11.1	−2.2	0.734
Protein, g/d^−1^	79.2 ± 2.8	76.6 ± 6.9	−2.6	70.0 ± 3.6	63.2 ± 2.1	−6.8	68.8 ± 7.2	72.2 ± 3.2	+3.4	0.498
Fat, g/d^−1^	65.2 ± 4.7	63.7 ± 4.1	−1.5	57.9 ± 3.9	57.0 ± 3.8	−0.9	64.1 ± 7.1	66.1 ± 4.7	+2.0	0.934
**Anthropometry**										
Weight, kg	82.8 ± 2.1	83.1 ± 2.0	+0.3	83.9 ± 2.5	84.1 ± 2.6	+0.2	85.7 ± 6.2	85.2 ± 6.3	−0.5	0.339
Fat Mass, kg	20.3 ± 0.8	19.4 ± 0.7	−0.9*	20.5 ± 1.0	20.1 ± 1.1	−0.4	23.8 ± 3.2	22.8 ± 3.2	−1.0*	0.279
Lean Mass, kg	62.5 ± 1.9	63.7 ± 1.8	+1.2*	63.4 ± 1.5	64.0 ± 1.6	+0.6*	61.9 ± 3.2	62.4 ± 3.2	+0.5	0.186
VAT Area, cm^−3^	104.5 ± 8.3	101.9 ± 8.1	−2.6	118.0 ± 11.5	121.2 ± 8.3	+2.2	116.7 ± 16.9	132.7 ± 12.4	+16.0	0.567
SAT Area, cm^−3^	206.8 ± 12.6	190.7 ± 8.6	−16.1	213.5 ± 16.7	217.2 ± 12.0	+3.7	231.7 ± 26.0	228.2 ± 26.6	−3.5	0.359

Values are presented as the group means ± SE. *Significant changes (*P* < 0.05) within group.

Abbreviations: RT, resistance training; HIAT, high-intensity aerobic training; MICT, moderate-intensity continuous training. TEI, total daily energy intake; Carbo, Carbohydrate VAT, visceral adipose tissue; SAT, subcutaneous adipose tissue.

**Table 4:** The Outcomes of Liver Function Test, Apoptosis, Inflammation and Oxidative stress, Insulin Resistance and Lipid Profile Values in 52 Subjects with NAFLD Who were Randomly Allocated to One of Three 12-week Exercise Training Programs.

	RT			HIAT			MICT			*P*
	n = 19			n = 20			n = 13			
	Baseline	After	Change	Baseline	After	Change	Baseline	After	Change	
**Liver Function test**										
^a^_Log_AST, U·L^−1^	1.365 ± 0.022	1.381 ± 0.020	+0.016	1.496 ± 0.048	1.476 ± 0.042	−0.020	1.346 ± 0.034	1.332 ± 0.035	−0.014	0.579
^a^ALT, U·L^−1^	29.2 ± 2.0	25.7 ± 2.0	−3.5	57.1 ± 11.2	50.9 ± 9.1	−6.2	32.2 ± 5.8	26.5 ± 4.4	−5.7*	0.215
_Log_γGTP, U·L^−1^	1.591 ± 0.07	1.510 ± 0.05	−0.081*	1.668 ± 0.050	1.627 ± 0.054	−0.041*	1.566 ± 0.078	1.505 ± 0.093	−0.061*	0.429
**Insulin Resistance and Lipid Profile**										
^a^_Log_FPG	1.991 ± 0.010	1.990 ± 0.015	−0.001	2.009 ± 0.008	2.003 ± 0.013	−0.006	1.947 ± 0.021	1.962 ± 0.006	+0.015	0.416
^a^HOMA-IR	2.00 ± 0.24	1.88 ± 0.25	−0.12	3.45 ± 0.50	3.25 ± 0.48	−0.20	2.24 ± 0.37	2.18 ± 0.29	−0.06	0.637
_Log_Triglycerides	2.042 ± 0.061	2.058 ± 0.062	+0.016	2.116 ± 0.048	2.091 ± 0.041	−0.025	2.179 ± 0.086	2.109 ± 0.069	−0.070	0.326
FFAs, Eq·L^−1^	0.59 ± 0.05	0.45 ± 0.04	−0.14*	0.57 ± 0.05	0.49 ± 0.04	−0.08	0.75 ± 0.11	0.47 ± 0.04	−0.28*	0.162
**Apoptosis, Inflammation and Oxidative Stress**										
^a^_Log_M30	2.143 ± 0.041	2.136 ± 0.029	−0.007	2.518 ± 0.080	2.479 ± 0.081	−0.039	2.177 ± 0.059	2.164 ± 0.048	−0.013	0.755
LPS, EU·mL^−1^	6.41 ± 0.64	7.94 ± 0.81	+1.53*	6.21 ± 0.63	6.80 ± 0.79	+0.59	4.24 ± 0.78	6.11 ± 0.98	+1.87*	0.424
Ferritin, μg·L^−1^	158.7 ± 30.7	138.4 ± 26.8	−20.3	208.5 ± 31.0	178.9 ± 26.9	−29.6*	143.8 ± 37.1	130.2 ± 32.5	−13.6	0.472
_Log_TBARS	3.750 ± 0.027	3.730 ± 0.034	−0.020	3.808 ± 0.021	3.812 ± 0.019	+0.004	3.799 ± 0.040	3.750 ± 0.036	−0.049	0.289

Values are presented as the group means ± SE. *Significant changes (*P* < 0.05) within group. ^a^ANCOVA with adjustments for respective baseline values were applied to compare changed values between groups.

Abbreviations: RT, resistance training; HIAT, high-intensity aerobic training; MICT, moderate-intensity continuous training; AST, aspartate aminotransferase; ALT, alanine aminotransferase; γGT, gamma glutamyl transpeptidase; FPG, fasting plasma glucose; HOMA-IR, insulin resistance by homeostasis model; FFAs, free fatty acids; LPS, lipopolysaccharide; TBARS, thiobarbituric acid reactive substances.

**Table 5:** Expression Levels of Fat Metabolism-related Genes, Macrophage Specific Genes and Nrf2 Target Genes in PBMC for 52 Subjects with NAFLD Who were Randomly Allocated to One of Three 12-week Exercise Training Programs.

	RT (n = 19)			HIAT (n = 20)			MICT (n = 13)			*P*
	Baseline	After	Change	Baseline	After	Change	Baseline	After	Change	
**Genes Involved in Fat Synthesis**										
_Log_SREBP1c	1.549 ± 0.097	1.380 ± 0.083	−0.169*	1.687 ± 0.095	1.450 ± 0.080	−0.237*	1.580 ± 0.131	1.416 ± 0.111	−0.164*	0.356
_Log_ACC	2.123 ± 0.233	2.024 ± 0.230	−0.099*	2.462 ± 0.245	2.319 ± 0.246	−0.143*	2.260 ± 0.312	2.150 ± 0.309	−0.110*	0.991
**Genes Involved in Fat Degradation**										
_Log_CPT1	1.529 ± 0.093	1.384 ± 0.085	−0.145	1.564 ± 0.090	1.432 ± 0.082	−0.132	1.660 ± 0.124	1.442 ± 0.113	−0.218*	0.799
_Log_ACO	1.533 ± 0.097	1.329 ± 0.080	−0.204*	1.586 ± 0.094	1.393 ± 0.078	−0.193*	1.583 ± 0.130	1.417 ± 0.107	−0.166*	0.935
**Macrophage Specific Gene**										
_Log_CD11b	1.491 ± 0.108	1.210 ± 0.101	−0.281*	1.649 ± 0.105	1.374 ± 0.099	−0.275*	1.568 ± 0.145	1.309 ± 0.136	−0.259*	0.964
_Log_CD14	1.670 ± 0.090	1.447 ± 0.090	−0.223*	1.761 ± 0.087	1.534 ± 0.087	−0.227*	1.696 ± 0.121	1.520 ± 0.120	−0.176*	0.516
_Log_CD68	1.578 ± 0.091	1.467 ± 0.090	−0.111*	1.685 ± 0.088	1.565 ± 0.088	−0.120*	1.630 ± 0.122	1.518 ± 0.121	−0.112*	0.969
_Log_TLR4	1.407 ± 0.091	1.199 ± 0.073	−0.208*	1.438 ± 0.089	1.259 ± 0.072	−0.179*	1.420 ± 0.123	1.239 ± 0.099	−0.181*	0.941
**Nrf2 Target Gene**										
_Log_HO1	2.085 ± 0.031	2.095 ± 0.040	+0.010	2.082 ± 0.033	2.143 ± 0.046	+0.061*	2.141 ± 0.035	2.140 ± 0.046	−0.001	0.855
_Log_NQO1	2.027 ± 0.044	1.977 ± 0.048	−0.050	1.953 ± 0.039	1.910 ± 0.044	−0.043	1.947 ± 0.047	1.952 ± 0.052	+0.005	0.643

Values are presented as the group means ± standard error. *Significant changes (*P* < 0.05) within group. Abbreviations: PBMC, peripheral blood mononuclear cell; RT, resistance training; HIAT, high-intensity aerobic training; MICT, moderate-intensity continuous training; SREBP1c, sterol regulatory element-binding protein 1c; ACC, acetyl-CoA carboxylase; CPT1, carnitine palmitoyltransferase I; ACO, acyl CoA oxidase; CD, cluster of differentiation; TLR, toll-like receptor; Nrf2, nuclear factor E2–related factor 2; HO1, heme oxygenase; NQO1, NADH quinone oxidoreductase.
